# Supplementary material for: Wheelchair service provision education in Canadian occupational therapy programs
Source: PLoS One. 2022 Feb 17;17(2):e0262165. doi: 10.1371/journal.pone.0262165 (PMC8853462; doi:10.1371/journal.pone.0262165)
Supplement: S1 File — (PDF) [file pone.0262165.s001.pdf]

ENHANCING WHEELCHAIR EDUCATION IN CANADIAN OCCUPATIONAL THERAPY UNIVERSITY PROGRAMS

Objective 1: Sociodemographic Form

1. What is your gender?
   - ☐ Man
   - ☐ Woman
   - ☐ Non-binary
   - ☐ Prefer not to answer

2. What is your age? ________

3. What is your primary language?
   - ☐ English
   - ☐ French
   - ☐ Other:______________

4. What is your level of education? (please check all that apply)
   - ☐ Diploma (OT)
   - ☐ Bachelors (OT)
   - ☐ Bachelors (other)
   - ☐ Masters (OT)
   - ☐ Masters (other)
   - ☐ Masters (research-based)
   - ☐ PhD

5. What is your position within the occupational therapy program?
   - ☐ Full-time faculty member
   - ☐ Part-time faculty member
   - ☐ Adjunct Faculty
   - ☐ Invited Presenter
   - ☐ Other: __________________

6. For how many years have you been a professor, clinical faculty, adjunct faculty or an invited presenter, in this occupational therapy program? _______

7. Do you currently practice clinically?
   - ☐ No
   - ☐ Yes

   In what area of practice? __________________________

   Does your current practice include wheelchair service provision? ☐ Yes ☐ No

8. What role(s) do you currently play in the delivery of wheelchair content in your program? (check all that apply)

- ☐ Coordinate a course that is exclusively wheelchair-specific
- ☐ Coordinate a course that includes wheelchair-specific content
- ☐ Teach within a course that is exclusively wheelchair-specific
- ☐ Teach wheelchair-specific content within a course
- ☐ Assist within a course that is exclusively wheelchair-specific
- ☐ Assist with wheelchair-specific content within a course
- ☐ Other wheelchair-relevant involvement (describe:)

9. For how many years have you been teaching wheelchair content in the occupational therapy program? __________

10. Have you developed, or contributed to the development, of a wheelchair-specific course or module for use within the occupational therapy program?
- ☐ No
- ☐ Yes

11. Since completion of your clinical degree, have you taken additional courses specific to wheelchair service provision?
- ☐ No
- ☐ Yes (please check all that apply)
  - o Continuing Education offered through a university program
  - o Conference workshop
  - o Educational opportunity offered by a private organization
  - o Online course (ex: MOOC)
  - o Self-study
  - o Other: ________________________

12. Do you have an additional certification related to wheelchair service provision? (Ex: RESNA Seating and Mobility Specialist Certification, ISWP certification)
- ☐ No
- ☐ Yes (please specify certification) ______________________

13. Are you a member of an organization or community of practice that is wheelchair-related?
- ☐ No
- ☐ Yes
  - ☐ RESNA
  - ☐ ISWP
  - ☐ ISPO
  - ☐ Other (describe:)
  - ☐ Other (describe:)
