# Supplementary material for: Wheelchair service provision education in Canadian occupational therapy programs
Source: PLoS One. 2022 Feb 17;17(2):e0262165. doi: 10.1371/journal.pone.0262165 (PMC8853462; doi:10.1371/journal.pone.0262165)
Supplement: S2 File — (PDF) [file pone.0262165.s002.pdf]

ENHANCING WHEELCHAIR EDUCATION IN CANADIAN OCCUPATIONAL THERAPY UNIVERSITY PROGRAMS

Section 1: University Program Descriptives
In this section, we are gathering demographic data on your occupational therapy program.
Q1. Which university do you represent?

- ☐ University of British Columbia
- ☐ University of Alberta
- ☐ University of Manitoba
- ☐ University of Toronto
- ☐ Western University
- ☐ McMaster University
- ☐ Queens University
- ☐ Université de Ottawa
- ☐ Université de Montréal
- ☐ McGill University
- ☐ Université Laval
- ☐ Université de Sherbrooke
- ☐ Université du Québec à Trois-Rivières
- ☐ Dalhousie University

Q2. What is the current entry-level degree of the occupational therapy program?

- ☐ M. Sc. (OT)
- ☐ M. Erg.
- ☐ M. Sc. Health (OT)
- ☐ M. OT.
- ☐ M. Sc. A. (OT)
- ☐ Other: ________________

Q3. What is the length of your program? __________

Q4. How many students are admitted each year? ___________

Q5. What is the primary language in which your program is taught?

- ☐ English
- ☐ French

Section 2: Wheelchair Educators
In this section, we would like to learn about who is developing and teaching the wheelchair content in your program.

Q6. How many people contribute to the development and/or teaching of wheelchair content in your program? ________

Q7. How many people are participating in completion of this survey? ________

Section 3: Wheelchair Service Provision Content, Instructional Method and Evaluation of Student's Knowledge and Skills

In this section, we would like to learn about the wheelchair content that is taught in your program. Specifically, we would like to know what content is taught related to each of the 8 steps of wheelchair service delivery recommended by the World Health Organization (WHO), how it is taught, and how it is evaluated. Click **here** to see a table listing the 8 steps. For more detailed information regarding the WHO wheelchair service provision process, click on ***Guidelines on the Provision of Manual Wheelchairs in Less Resourced Settings*** and ***Wheelchair Service Training Package – Basic Level***. The WHO definition of each of the 8 steps may be found by hovering over the name of the step itself in the questions below. Note: Content related to the 8 steps might be included in a variety of ways (e.g., lecture; class notes; integration in a problem-based learning case study; a course dedicated to wheelchair content).

Q8. Does your program include content related to *Referral and Appointment*?

- ☐ Yes
- ☐ No

If yes:

a) Is it taught:

- ☐ in one consolidated **optional** course dedicated to wheelchair provision?
  i. If yes, where does this course reside within the curriculum?
    - ☐ Year 1
    - ☐ Year 2
    - ☐ Year 3
    - ☐ Year 4
- ☐ in one consolidated **required** course dedicated to wheelchair provision?
  i. If yes, where does this course reside within the curriculum?
    - ☐ Year 1
    - ☐ Year 2
    - ☐ Year 3
    - ☐ Year 4
- ☐ in a module or portion of another course?
  i. If yes, where does this course reside within the curriculum?
    - ☐ Year 1
    - ☐ Year 2
    - ☐ Year 3
    - ☐ Year 4
- ☐ in a number of courses throughout the program?
  i. If yes, in how many courses? ________
  ii. If yes, where do these courses reside within the curriculum? (check all that apply)
    - ☐ Year 1
    - ☐ Year 2
    - ☐ Year 3
    - ☐ Year 4

iii. Approximately how many hours are dedicated to *Referral and Appointment*?

b) Do you use content from (check all that apply):

- ☐ External resources developed outside of your program (e.g., including but not limited to published articles, programs, position papers, guidelines or 'in-house' assessment forms from a local rehabilitation centre)? Please list / describe the resources that you use. _______________
- ☐ Content developed internally within your program, informed by external resources (e.g., a module based on an external resource)? Please describe resources developed: _______________. Please list / describe the external resources (e.g., published articles, programs, position papers, guidelines) used. ____

c) Which of the following methods do you use to teach this content? (check all that apply)

- ☐ lecture
- ☐ practical lab with demonstration from instructors
- ☐ practical lab with demonstration from instructors + simulated clients
- ☐ practical lab with demonstration from instructors + actual wheelchair users
- ☐ online module
- ☐ a hybrid approach (lecture / lab / online)
- ☐ other: ________________

d) Does this component of the wheelchair service delivery process fit within the scope of practice of occupational therapists in your region?

- ☐ Yes
- ☐ No

e) Which of the following methods do you use to evaluate your students' knowledge and skills related to *Referral and Appointment*? (check all that apply)

- ☐ Written evaluation
- ☐ Practical evaluation
- ☐ Online evaluation
- ☐ Other:

Q9. Does your program include content related to *Assessment*?

- ☐ Yes
- ☐ No

If yes:

a) Is it taught:

- ☐ in one consolidated **optional** course dedicated to wheelchair provision?
  - i. If yes, where does this course reside within the curriculum?
    - ☐ Year 1
    - ☐ Year 2

- ☐ Year 3
- ☐ Year 4

☐ in one consolidated **required** course dedicated to wheelchair provision?

i. If yes, where does this course reside within the curriculum?

- ☐ Year 1
- ☐ Year 2
- ☐ Year 3
- ☐ Year 4

☐ in a module or portion of another course?

i. If yes, where does this course reside within the curriculum?

- ☐ Year 1
- ☐ Year 2
- ☐ Year 3
- ☐ Year 4

☐ in a number of courses throughout the program?

i. If yes, in how many courses? _______

ii. If yes, where do these courses reside within the curriculum? (check all that apply)

- ☐ Year 1
- ☐ Year 2
- ☐ Year 3
- ☐ Year 4

iii. Approximately how many hours are dedicated to *Assessment*?

b) Do you use content from (check all that apply):

- ☐ External resources developed outside of your program (e.g., including but not limited to published articles, programs, position papers, guidelines or 'in-house' assessment forms from a local rehabilitation centre)? Please list / describe the resources that you use. _______________
- ☐ Content developed internally within your program, informed by external resources (e.g., a module based on an external resource)? Please describe resources developed: _______________. Please list / describe the external resources (e.g., published articles, programs, position papers, guidelines) used. ____

c) Which of the following methods do you use to teach this content? (check all that apply)

- ☐ lecture
- ☐ practical lab with demonstration from instructors
- ☐ practical lab with demonstration from instructors + simulated clients
- ☐ practical lab with demonstration from instructors + actual wheelchair users
- ☐ online module
- ☐ a hybrid approach (lecture / lab / online)
- ☐ other: ________________

d) Does this component of the wheelchair service delivery process fit within the scope of practice of occupational therapists in your region?

- ☐ Yes
- ☐ No

e) Which of the following methods do you use to evaluate your students' knowledge and skills related to *Assessment*? (check all that apply)

- ☐ Written evaluation
- ☐ Practical evaluation
- ☐ Online evaluation
- ☐ Other:

Q10. Does your program include content related to *Prescription*?

- ☐ Yes
- ☐ No

If yes:

a) Is it taught:

- ☐ in one consolidated **optional** course dedicated to wheelchair provision?
  - i. If yes, where does this course reside within the curriculum?
    - ☐ Year 1
    - ☐ Year 2
    - ☐ Year 3
    - ☐ Year 4
- ☐ in one consolidated **required** course dedicated to wheelchair provision?
  - i. If yes, where does this course reside within the curriculum?
    - ☐ Year 1
    - ☐ Year 2
    - ☐ Year 3
    - ☐ Year 4
- ☐ in a module or portion of another course?
  - i. If yes, where does this course reside within the curriculum?
    - ☐ Year 1
    - ☐ Year 2
    - ☐ Year 3
    - ☐ Year 4
- ☐ in a number of courses throughout the program?
  - i. If yes, in how many courses? ________
  - ii. If yes, where do these courses reside within the curriculum? (check all that apply)
    - ☐ Year 1
    - ☐ Year 2
    - ☐ Year 3
    - ☐ Year 4
  - iii. Approximately how many hours are dedicated to *Prescription*?

b) Do you use content from (check all that apply):

- ☐ External resources developed outside of your program (e.g., including but not limited to published articles, programs, position papers, guidelines or 'in-house' assessment forms from a local rehabilitation centre)? Please list / describe the resources that you use. _______________
- ☐ Content developed internally within your program, informed by external resources (e.g., a module based on an external resource)? Please describe resources developed: _______________. Please list / describe the external resources (e.g., published articles, programs, position papers, guidelines) used. ____

c) Which of the following methods do you use to teach this content? (check all that apply)

- ☐ lecture
- ☐ practical lab with demonstration from instructors
- ☐ practical lab with demonstration from instructors + simulated clients
- ☐ practical lab with demonstration from instructors + actual wheelchair users
- ☐ online module
- ☐ a hybrid approach (lecture / lab / online)
- ☐ other: ________________

d) Does this component of the wheelchair service delivery process fit within the scope of practice of occupational therapists in your region?

- ☐ Yes
- ☐ No

e) Which of the following methods do you use to evaluate your students' knowledge and skills related to *Prescription*? (check all that apply)

- ☐ Written evaluation
- ☐ Practical evaluation
- ☐ Online evaluation
- ☐ Other:

Q11. Does your program include content related to *Funding and Ordering*?

- ☐ Yes
- ☐ No

If yes:

a) Is it taught:

- ☐ in one consolidated **optional** course dedicated to wheelchair provision?
  - i. If yes, where does this course reside within the curriculum?
    - ☐ Year 1
    - ☐ Year 2
    - ☐ Year 3
    - ☐ Year 4
- ☐ in one consolidated **required** course dedicated to wheelchair provision?

i. If yes, where does this course reside within the curriculum?
- ☐ Year 1
- ☐ Year 2
- ☐ Year 3
- ☐ Year 4

☐ in a module or portion of another course?

i. If yes, where does this course reside within the curriculum?
- ☐ Year 1
- ☐ Year 2
- ☐ Year 3
- ☐ Year 4

☐ in a number of courses throughout the program?

i. If yes, in how many courses? _______

ii. If yes, where do these courses reside within the curriculum? (check all that apply)
- ☐ Year 1
- ☐ Year 2
- ☐ Year 3
- ☐ Year 4

iii. Approximately how many hours are dedicated to *Funding and Ordering*?

b) Do you use content from (check all that apply):

- ☐ External resources developed outside of your program (e.g., including but not limited to published articles, programs, position papers, guidelines or 'in-house' assessment forms from a local rehabilitation centre)? Please list / describe the resources that you use. _______________
- ☐ Content developed internally within your program, informed by external resources (e.g., a module based on an external resource)? Please describe resources developed: _______________. Please list / describe the external resources (e.g., published articles, programs, position papers, guidelines) used. ____

c) Which of the following methods do you use to teach this content? (check all that apply)

- ☐ lecture
- ☐ practical lab with demonstration from instructors
- ☐ practical lab with demonstration from instructors + simulated clients
- ☐ practical lab with demonstration from instructors + actual wheelchair users
- ☐ online module
- ☐ a hybrid approach (lecture / lab / online)
- ☐ other: ________________

d) Does this component of the wheelchair service delivery process fit within the scope of practice of occupational therapists in your region?

- ☐ Yes
- ☐ No

e) Which of the following methods do you use to evaluate your students' knowledge and skills related to *Funding and Ordering*? (check all that apply)

- ☐ Written evaluation
- ☐ Practical evaluation
- ☐ Online evaluation
- ☐ Other:

Q12. Does your program include content related to *Product Preparation*?

- ☐ Yes
- ☐ No

If yes:

a) Is it taught:

- ☐ in one consolidated **optional** course dedicated to wheelchair provision?
  - i. If yes, where does this course reside within the curriculum?
    - ☐ Year 1
    - ☐ Year 2
    - ☐ Year 3
    - ☐ Year 4
- ☐ in one consolidated **required** course dedicated to wheelchair provision?
  - i. If yes, where does this course reside within the curriculum?
    - ☐ Year 1
    - ☐ Year 2
    - ☐ Year 3
    - ☐ Year 4
- ☐ in a module or portion of another course?
  - i. If yes, where does this course reside within the curriculum?
    - ☐ Year 1
    - ☐ Year 2
    - ☐ Year 3
    - ☐ Year 4
- ☐ in a number of courses throughout the program?
  - i. If yes, in how many courses? ________
  - ii. If yes, where do these courses reside within the curriculum? (check all that apply)
    - ☐ Year 1
    - ☐ Year 2
    - ☐ Year 3
    - ☐ Year 4
  - iii. Approximately how many hours are dedicated to *Product Preparation*?

b) Do you use content from (check all that apply):

- External resources developed outside of your program (e.g., including but not limited to published articles, programs, position papers, guidelines or 'in-house' assessment forms from a local rehabilitation centre)? Please list / describe the resources that you use. ________________
- Content developed internally within your program, informed by external resources (e.g., a module based on an external resource)? Please describe resources developed: ________________. Please list / describe the external resources (e.g., published articles, programs, position papers, guidelines) used. ____

c) Which of the following methods do you use to teach this content? (check all that apply)

- lecture
- practical lab with demonstration from instructors
- practical lab with demonstration from instructors + simulated clients
- practical lab with demonstration from instructors + actual wheelchair users
- online module
- a hybrid approach (lecture / lab / online)
- other: _________________

d) Does this component of the wheelchair service delivery process fit within the scope of practice of occupational therapists in your region?

- Yes
- No

e) Which of the following methods do you use to evaluate your students' knowledge and skills related to *Product Preparation*? (check all that apply)

- Written evaluation
- Practical evaluation
- Online evaluation
- Other:

Q13. Does your program include content related to *Fitting*?

- Yes
- No

If yes:

a) Is it taught:

- in one consolidated **optional** course dedicated to wheelchair provision?
  i. If yes, where does this course reside within the curriculum?
    - Year 1
    - Year 2
    - Year 3
    - Year 4
- in one consolidated **required** course dedicated to wheelchair provision?
  i. If yes, where does this course reside within the curriculum?

- ☐ Year 1
- ☐ Year 2
- ☐ Year 3
- ☐ Year 4

☐ in a module or portion of another course?

i. If yes, where does this course reside within the curriculum?
- ☐ Year 1
- ☐ Year 2
- ☐ Year 3
- ☐ Year 4

☐ in a number of courses throughout the program?

i. If yes, in how many courses? _______

ii. If yes, where do these courses reside within the curriculum? (check all that apply)
- ☐ Year 1
- ☐ Year 2
- ☐ Year 3
- ☐ Year 4

iii. Approximately how many hours are dedicated to *Fitting*?

b) Do you use content from (check all that apply):

☐ External resources developed outside of your program (e.g., including but not limited to published articles, programs, position papers, guidelines or 'in-house' assessment forms from a local rehabilitation centre)? Please list / describe the resources that you use. _______________

☐ Content developed internally within your program, informed by external resources (e.g., a module based on an external resource)? Please describe resources developed: _______________. Please list / describe the external resources (e.g., published articles, programs, position papers, guidelines) used. ____

c) Which of the following methods do you use to teach this content? (check all that apply)

☐ lecture
☐ practical lab with demonstration from instructors
☐ practical lab with demonstration from instructors + simulated clients
☐ practical lab with demonstration from instructors + actual wheelchair users
☐ online module
☐ a hybrid approach (lecture / lab / online)
☐ other: ________________

d) Does this component of the wheelchair service delivery process fit within the scope of practice of occupational therapists in your region?

☐ Yes
☐ No

e) Which of the following methods do you use to evaluate your students' knowledge and skills related to *Fitting*? (check all that apply)

- ☐ Written evaluation
- ☐ Practical evaluation
- ☐ Online evaluation
- ☐ Other:

Q14. Does your program include content related to *User Training*?

- ☐ Yes
- ☐ No

If yes:

a) Is it taught:

- ☐ in one consolidated **optional** course dedicated to wheelchair provision?
  - i. If yes, where does this course reside within the curriculum?
    - ☐ Year 1
    - ☐ Year 2
    - ☐ Year 3
    - ☐ Year 4
- ☐ in one consolidated **required** course dedicated to wheelchair provision?
  - i. If yes, where does this course reside within the curriculum?
    - ☐ Year 1
    - ☐ Year 2
    - ☐ Year 3
    - ☐ Year 4
- ☐ in a module or portion of another course?
  - i. If yes, where does this course reside within the curriculum?
    - ☐ Year 1
    - ☐ Year 2
    - ☐ Year 3
    - ☐ Year 4
- ☐ in a number of courses throughout the program?
  - i. If yes, in how many courses? ________
  - ii. If yes, where do these courses reside within the curriculum? (check all that apply)
    - ☐ Year 1
    - ☐ Year 2
    - ☐ Year 3
    - ☐ Year 4
  - iii. Approximately how many hours are dedicated to *User Training*?

b) Do you use content from (check all that apply):

- ☐ External resources developed outside of your program (e.g., including but not limited to published articles, programs, position papers, guidelines or 'in-

house' assessment forms from a local rehabilitation centre)? Please list / describe the resources that you use. _______________

- ☐ Content developed internally within your program, informed by external resources (e.g., a module based on an external resource)? Please describe resources developed: _______________. Please list / describe the external resources (e.g., published articles, programs, position papers, guidelines) used. ____

c) Which of the following methods do you use to teach this content? (check all that apply)

- ☐ lecture
- ☐ practical lab with demonstration from instructors
- ☐ practical lab with demonstration from instructors + simulated clients
- ☐ practical lab with demonstration from instructors + actual wheelchair users
- ☐ online module
- ☐ a hybrid approach (lecture / lab / online)
- ☐ other: ________________

d) Does this component of the wheelchair service delivery process fit within the scope of practice of occupational therapists in your region?

- ☐ Yes
- ☐ No

e) Which of the following methods do you use to evaluate your students' knowledge and skills related to *User Training*? (check all that apply)

- ☐ Written evaluation
- ☐ Practical evaluation
- ☐ Online evaluation
- ☐ Other:

Q15. Does your program include content related to *Follow Up, Maintenance and Repairs*?

- ☐ Yes
- ☐ No

If yes:

a) Is it taught:

- ☐ in one consolidated **optional** course dedicated to wheelchair provision?
  - i. If yes, where does this course reside within the curriculum?
    - ☐ Year 1
    - ☐ Year 2
    - ☐ Year 3
    - ☐ Year 4
- ☐ in one consolidated **required** course dedicated to wheelchair provision?
  - i. If yes, where does this course reside within the curriculum?
    - ☐ Year 1
    - ☐ Year 2

- ☐ Year 3
  - ☐ Year 4
- ☐ in a module or portion of another course?
  i. If yes, where does this course reside within the curriculum?
    - ☐ Year 1
    - ☐ Year 2
    - ☐ Year 3
    - ☐ Year 4
- ☐ in a number of courses throughout the program?
  i. If yes, in how many courses? _______
  ii. If yes, where do these courses reside within the curriculum? (check all that apply)
    - ☐ Year 1
    - ☐ Year 2
    - ☐ Year 3
    - ☐ Year 4
  iii. Approximately how many hours are dedicated to *Follow Up, Maintenance and Repairs*?

b) Do you use content from (check all that apply):

- ☐ External resources developed outside of your program (e.g., including but not limited to published articles, programs, position papers, guidelines or 'in-house' assessment forms from a local rehabilitation centre)? Please list / describe the resources that you use. _______________
- ☐ Content developed internally within your program, informed by external resources (e.g., a module based on an external resource)? Please describe resources developed: _______________. Please list / describe the external resources (e.g., published articles, programs, position papers, guidelines) used. ____

c) Which of the following methods do you use to teach this content? (check all that apply)

- ☐ lecture
- ☐ practical lab with demonstration from instructors
- ☐ practical lab with demonstration from instructors + simulated clients
- ☐ practical lab with demonstration from instructors + actual wheelchair users
- ☐ online module
- ☐ a hybrid approach (lecture / lab / online)
- ☐ other: ________________

d) Does this component of the wheelchair service delivery process fit within the scope of practice of occupational therapists in your region?

- ☐ Yes
- ☐ No

e) Which of the following methods do you use to evaluate your students' knowledge and skills related to *Follow Up, Maintenance and Repairs*? (check all that apply)

- ☐ Written evaluation
- ☐ Practical evaluation
- ☐ Online evaluation
- ☐ Other:

Q16. Outside of course-related evaluation, do you collect any data or information about your students' learning (ex: outcome measures such as the Wheelchair Skills Test or the International Society of Wheelchair Professionals Basic Test) or your student's perceptions regarding the wheelchair service provision education provided?

- ☐ No
- ☐ Yes

If yes, please describe how you have measured it. ___________
