# Supplementary material for: Wheelchair service provision education in Canadian occupational therapy programs
Source: PLoS One. 2022 Feb 17;17(2):e0262165. doi: 10.1371/journal.pone.0262165 (PMC8853462; doi:10.1371/journal.pone.0262165)
Supplement: Attachment — Submitted filename: Response to Reviewers.docx [file pone.0262165.s004.docx]

**Response to Reviewers**

Dear Reviewers:

Thank you for the constructive and comprehensive feedback on our manuscript. We have outlined the feedback comments individually in the table below, along with our response and/or revision for each item.

| **Item** | **Response** |
| --- | --- |
| R1. In the abstract in line 28 I advise not to specify the 8 phases because there is no correspondence with the introduction. | Thank you for this. We have removed “8-steps” from this sentence (line 28). |
| R1. Explain better from line 131 to line 133 (it is not clear) | This sentence was revised to improve comprehension (lines 132-134). |
| R1. Line 136-139 should belong to section 1, you could insert it before | The questionnaire was separate from the University sociodemographic information, as it related to the respondents individually. We have revised this sentence to make it clearer (lines 137-138). |
| R1. In the materials and methods you could insert the questionnaire to explain better | Thank you for this suggestion. We have now included both the Sociodemographic Questionnaire and Educator Survey as Supporting Information with this revision (S1 and S2 Files). |
| R1. In discussion: from line 220 all numerical evaluations should be included in the results | The numerical values included here are all drawn directly from Table 2 (Total time column; Number of steps covered column). |
| R1. Bibliography should be included in the discussion to support the considerations | Thank you for this suggestion. In addition to the existing citations in paragraph 3 (2 references), paragraph 4 (6 references), and paragraph 5 (2 references), we have further incorporated the literature in support of our findings on lines 228-231, 255-257, and 293. |
| R2. - the small number of educators involved in comparison to the programs (less than 2 for each occupational therapy program) | Thank you for this observation. In recruiting for this study, we solicited individuals who taught this specific content area (via recommendation from each program director). In Canadian OT programs, wheelchair-related content is typically taught by a select number of faculty (or clinicians with this expertise are brought in); consequently, the total number of potential respondents aware of wheelchair-specific curricula is small. In our survey we inquired how many individuals contributed to development or teaching of this content and the total across programs was 67, so our participation was 43% of all individuals related in any way to content delivery, which we would consider to be substantial. We have added an acknowledgement of this sub-set in the limitations section (lines 305-308). |
| R2. the lack of the student perception about the theme investigated, both on the quality of the programs and the time dedicated to it. Evaluate the quality of the program only investigating the educators' side appears a partial overview | This is an interesting point. Obtaining student perspectives would provide another insight into wheelchair-related education. We acknowledge our intent was to obtain a quantitative description of content provision (related to the WHO 8-step structure) as an important first step, rather than assess the quality of education – which would indeed also be an important and interesting study. We have taken your suggestion and incorporated it as a useful future direction (lines 319-321). |
| R2. the conclusion section does not draw conclusive consideration about the data obtained but repeats the discussion and results | We appreciate your input on this. The conclusion has been reformulated to focus on what is novel and define the primary conclusions, as well as how this directs next steps and future research. |
| R2. the discussion and conclusions on the external validity of the results appears lacking | Thank you for this comment. We have added to the limitations section on the generalizability (external validity) of this study on lines 316-318. |
| R2. Title: the study design is missing | We have adapted the title to address this as follows: *A survey of wheelchair service provision education in Canadian occupational therapy programs* |
| R2. It is preferable to not use abbreviations in the abstract, except WHO that is worldwide known to all readers | Thank you for pointing this out. We have removed “OT” and used occupational therapy instead. |
| R2. the time necessary to complete the survey could be interesting to evaluate the educators' small number of complete answers | We were not able to obtain completion time data with our survey platform so it is difficult to make any conclusions about how this impacted responses. After pilot-testing, we indicated to participants that the survey would take 30-40 minutes to complete. As we had a (collective) response from each program and little missing data, we are optimistic that the length of the survey was not a deterrent. We have added in the estimated completion time (based on pilot testing) in the Measurement section on line 127-128. |
| R2. 29 educators could appear as a high number if the total number of possible educators engageable is not stated | As per the comment addressed earlier, we believe we obtained >40% of individuals involved in teaching this content area. We added a sentence in the discussion (lines 217-219) to elaborate on the point you have raised here. |
| R2. It seems unnecessary to report both in minutes and hours the time allocated to the programs; choose one of them | Thank you, this would simplify things. We have revised the sentence on Line 184 to read “*2.5 to 48 hours*” and Table 2, as well as in Lines 226 and 238-239. |
