# Supplementary material for: Wheelchair service provision education in Canadian occupational therapy programs
Source: PLoS One. 2022 Feb 17;17(2):e0262165. doi: 10.1371/journal.pone.0262165 (PMC8853462; doi:10.1371/journal.pone.0262165)
Supplement: Attachment — Submitted filename: Response to Reviewers.docx [file pone.0262165.s005.docx]

**Response to Reviewers**

Dear Dr. de Sire:

Thank you for your feedback on our manuscript. We have revised our paper to address the concerns as outlined below.

Please revise your Discussion in order to make the paper more intriguing and suitable for publication. I suggest you improve it, citing and discussing recent papers.

*We have made revisions to the Discussion section to improve its suitability. In addition to the existing citations, most of which are from 2019-2021, we have introduced four additional citations all of which were published in 2021 and integrated this into the content and application of our findings.*
